# The *DRD3* Ser9Gly Polymorphism Predicted Metabolic Change in Drug-Naive Patients With Bipolar II Disorder: Erratum

**DOI:** 10.1097/01.md.0000504574.73365.1b

**Published:** 2016-10-14

In the article “The *DRD3* Ser9Gly Polymorphism Predicted Metabolic Change in Drug-Naive Patients With Bipolar II Disorder”,^[1]^ which appeared in Volume 95, Issue 24 of *Medicine*, Dr. Ting-Ting Chang's affiliation was originally given incorrectly. The article has since been corrected online.
